# Prevalence of established and emerging biomarkers of immune checkpoint inhibitor response in advanced hepatocellular carcinoma

**DOI:** 10.18632/oncotarget.26998

**Published:** 2019-06-18

**Authors:** Celina Ang, Samuel J. Klempner, Siraj M. Ali, Russell Madison, Jeffrey S. Ross, Eric A. Severson, David Fabrizio, Aaron Goodman, Razelle Kurzrock, James Suh, Sherri Z. Millis

**Affiliations:** ^1^ Department of Medicine, Division of Hematology/Oncology, Tisch Cancer Institute, Mount Sinai Hospital, New York, NY, USA; ^2^ Department of Medicine, Division of Hematology/Oncology, Cedars-Sinai Medical Center, Los Angeles, CA, USA; ^3^ Foundation Medicine, Cambridge, MA, USA; ^4^ Department of Medicine, Division of Hematology and Oncology, Moores Cancer Center, University of California San Diego, San Diego, CA, USA

**Keywords:** immunotherapy, biomarkers, hepatocellular carcinoma, tumor mutation burden

## Abstract

The clinical deployment of immune checkpoint inhibitors (ICIs) has created a tandem drive for the identification of biomarkers linked to benefit. Comprehensive genomic profiling was performed to evaluate the frequency of genomic biomarkers of ICI response in 755 patients with advanced hepatocellular carcinoma (HCC). Median age was 62 years’ old, 73% were male, 46% had extrahepatic disease, 107 had documented hepatitis C, 96 had hepatitis B and 4 patients were coinfected. Median tumor mutation burden (TMB) was 4 mutations/Mb and only 6 tumors (0.8%) were TMB-high. Out of 542 cases assessed for microsatellite instability (MSI), one (0.2%) was MSI-high and TMB-high. Twenty-seven (4%) patients had *POLE/D* alterations. One patient had a pathogenic *POLE* R762W mutation but TMB was 4 mutations/Mb. Forty percent had DNA damage response gene alterations. In a small case series (N=17) exploring the relationship between biomarkers and ICI response, one patient (TMB 15 mutations/Mb, MSI-low) had a sustained complete response to nivolumab lasting > 2 years. Otherwise there were no significant genomic or TMB differences between responders, progressors, and those with stable disease. Overall, markers of genomic instability were infrequent in this cohort. Larger clinically annotated datasets are needed to explore genomic and non-genomic determinants of ICI response in HCC.

## INTRODUCTION

Virally associated cancers like hepatocellular carcinoma (HCC) illustrate the disrupted balance between immune system attack and regulation [1]. The immune checkpoints PD-1 and CTLA-4 are activated in the presence of a chronic infection to suppress cytotoxic T cells and prevent immune-mediated host tissue damage. Cancer cells commandeer and manipulate these regulatory signals to escape immune surveillance, resulting in unimpeded growth and metastasis. The concept of harnessing the immune system to attack cancer cells has been actualized with the development of immune checkpoint inhibitors (ICIs) which restore effector T cell function and cell mediated cytotoxicity. PD-1 inhibitors have shown activity across multiple solid and hematologic malignancies including HCC. Based on the results of the phase I/II CheckMate 040 and phase II KEYNOTE-224 trials [2, 3], nivolumab and pembrolizumab have been FDA approved for HCC after progression on sorafenib. Additional trials expanding the use of ICIs in HCC in earlier treatment contexts are underway, with the results of the phase III CheckMate 459 trial of sorafenib vs nivolumab in first-line eagerly anticipated.

Responses to ICIs occur in a limited subset of patients across most cancer types including HCC [4]. An unmet need exists to develop predictive biomarkers to identify individuals most likely to respond to immunotherapies. PD-1 ligand (i.e. PD-L1) expression is an FDA approved companion biomarker used to select patients with esophagogastric adenocarcinoma or non-small cell lung cancer suitable for treatment with PD-1 inhibitors. Mismatch repair protein (MMR) deficiency or microsatellite instability (MSI) is also an FDA approved indication for the use of PD-1 inhibitors in advanced solid tumors given the strikingly higher response rates observed in MMR deficient/MSI-high compared to MMR proficient or MSI-low tumors [5]. Other emerging biomarkers include tumor mutation burden (TMB), alterations in DNA damage response (DDR) genes and DNA polymerases epsilon and delta (*POLE* and *POLD*), among others.

Owing to historically lower biopsy rates and poor outcomes, few studies have examined the landscape of putative immunotherapy biomarkers in advanced HCC. In this study, we survey the genomic landscape of 755 HCC specimens to identify patterns and associations with current as well as emerging biomarkers of response to PD-1 inhibitors. We also include a case series of patients with advanced HCC who received PD-1 inhibitors and evaluate their genomic profiles in the context of their responses to these agents.

## RESULTS

### Patient characteristics

The median age of diagnosis was 62 years old (range 19-91 years). Female and male patients were 27% and 73% percent, respectively. Hepatitis B (HBV) infection was determined by chart review (96 noted/suggested HBV+), of which 84 were also HBV+ by sequencing. Chart review identified 111 patients with hepatitis C (HCV, 4 co-infected with HBV). Seven patients had nonalcoholic steatohepatitis (NASH), 1 had a history of alcoholic liver disease, and 54 had cirrhosis of undocumented etiology. Nearly half (47%) had documented metastastic disease, with the most common sites of metastases being lung (8%), bone (6%), abdominopelvic soft tissue (6%), lymph nodes (4%) and adrenal gland (2%). Patient characteristics are summarized in Table 1.

**Table 1:** Characteristics of study population and biomarker subgroups

Patient characteristics		Biomarker subgroups		
	Total *N* = 755	PD-L1+ *N* = 35	*POLE/D N* = 27	DDR *N* = 306
Median age (range)	62 (19-91)	65	67	62
% Female:male	27:73	29:71	33:67	27:73
Known etiology (*N* = 269)				
Hepatitis B	96	2	2	37
Hepatitis C	111^*^	9	7	42
NASH	7		1	4
Alcohol	1			
Cirrhosis etiology unknown	54	3	2	12
Known site of tissue tested		13 (37%)	15 (55%)	144 (47%)
Liver	513 (68%)	5	8	51
Lymph nodes	30 (4%)	1		14
Lung	60 (8%)	2	3	28
Bone	45 (6%)	2	2	21
Abdominopelvic soft tissue	45 (6%)	2	1	18
Adrenal gland	15 (2%)			4
Other	47 (6%)	1 gallbladder	1 ovary	4 brain, 1 ovary, 1kidney, 1 colon, 1 pancreas

^*^4 coinfected with Hepatitis B.

### Mutational landscape of HCC

**Figure 1:**
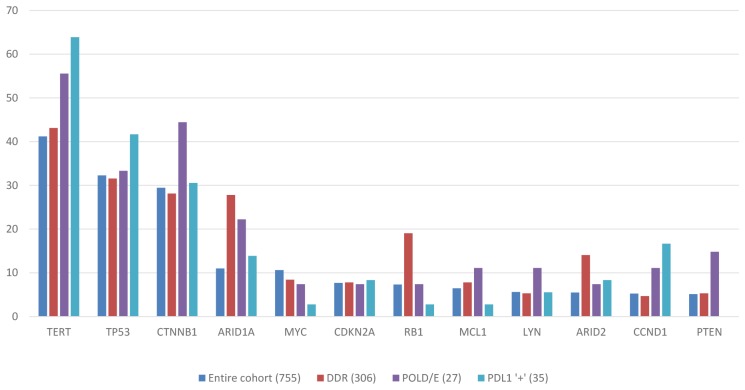
Mutational landscape of HCC across study cohort and biomarker subgroups.

Known or likely pathogenic mutations were identified in 751 cases; the other 4 cases only had variants of unknown significance (VUS). The most commonly altered genes were *TERT* (44%), *TP53* (35%), *CTNNB1* (31%), *ARID1A* (12%), and *MYC* (12%). All *MYC* alterations were amplifications. Mutations in *CDKN2A*, *RB1*, *ARID2*, *MCL1*, and *PTEN* occurred in 5-8% of specimens. Other alterations found at low rates that might be relevant for targeted therapies included *NTRK1* (2%), several PI3 kinase pathway genes (*TSC2, AKT3, STK11*), and *MET* (2–4%). No appreciable differences between the mutational profiles of primary HCCs and metastatic lesions were noted (data not shown). All alterations with a frequency >5% are shown in Figure 1.

### Tumor mutation burden (TMB), microsatellite status and PD-L1 expression

The median TMB for the entire cohort was 4 mutations/Mb, with 95% of cases having a TMB of < 10 mutations/Mb. Gender, HBV or HCV status, age and the proportion of primary vs metastatic tumors did not differ significantly amongst TMB subgroups (Table 2). Microsatellite status was determined in 542 specimens; only one was both MSI-high and TMB high (21 mutations/Mb). This specimen belonged to an 82 years’ old Caucasian male with unknown risk factors or cirrhosis status. Three (50%) tumors were MSI-low in the TMB-high group, 128 (68%) in the TMB intermediate and 411 (73%) in the TMB-low group (*p* > 0.05). Microsatellite status was ambiguous (ie. not MSI-low but below the MSI-high cutoff) in 4 specimens and not evaluable in 209 specimens.

**Table 2:** Tumor mutation burden (TMB) distribution by etiology

	TMB high	TMB intermediate	TMB low	All
***N* (%)**	6 (1%)	188 (25%)	561 (74%)	755
**% Male:Female**	67:33	75:25	72:28	73:27
**Median age**	58 years	63 years	61 years	62 years
**Specimen site**				
Primary liver/not noted	2 (33%)	94 (50%)	252 (45%)	348
Metastasis	4 (67%)	94 (50%)	309 (55%)	407
**Liver disease etiology**				
HCV (no HBV)	0	27 (25%)	80 (75%)	107
HBV (± HCV)	0	31 (32%)	65 (68%)	96
NASH	0	1(14%)	6 (86%)	7

PD-L1 expression levels were only available for 65 patients: 32 had “low positive”, 3 had “high positive” scores, and 29 were PD-L1 negative. PD-L1 positivity was not associated with high TMB; the 3 patients with high positive PD-L1 were TMB low (2-5 mutations/Mb). Gene alteration frequencies in PD-L1 positive tumors are shown in Figure 1.

**Figure 2:**
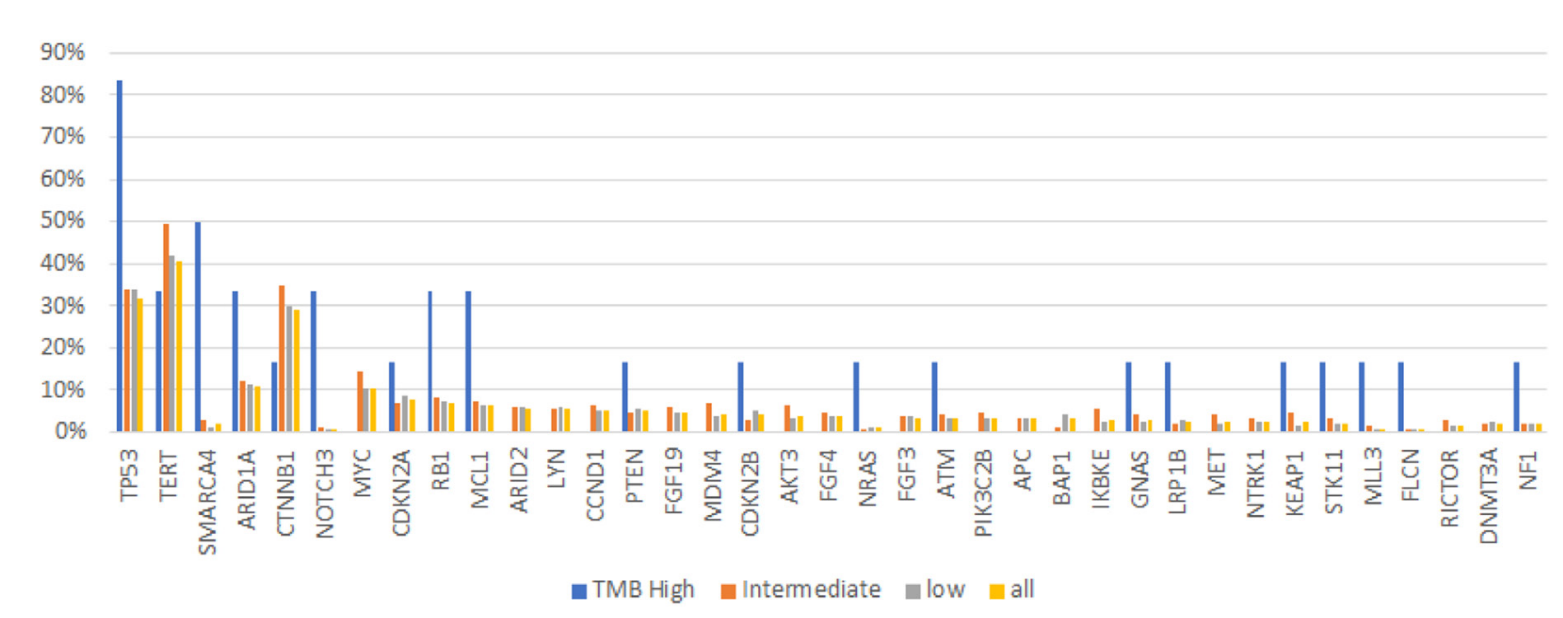
Differences in gene alteration frequencies by TMB level.

Differences in mutation profile amongst TMB subgroups were analyzed. Though TMB-high HCCs demonstrated some genetic differences compared to TMB-intermediate and low HCCs, their numbers were too small (*N*=6) to be conclusive. No significant differences emerged between TMB low and intermediate HCCs (Figure 2).

**Figure 3:**
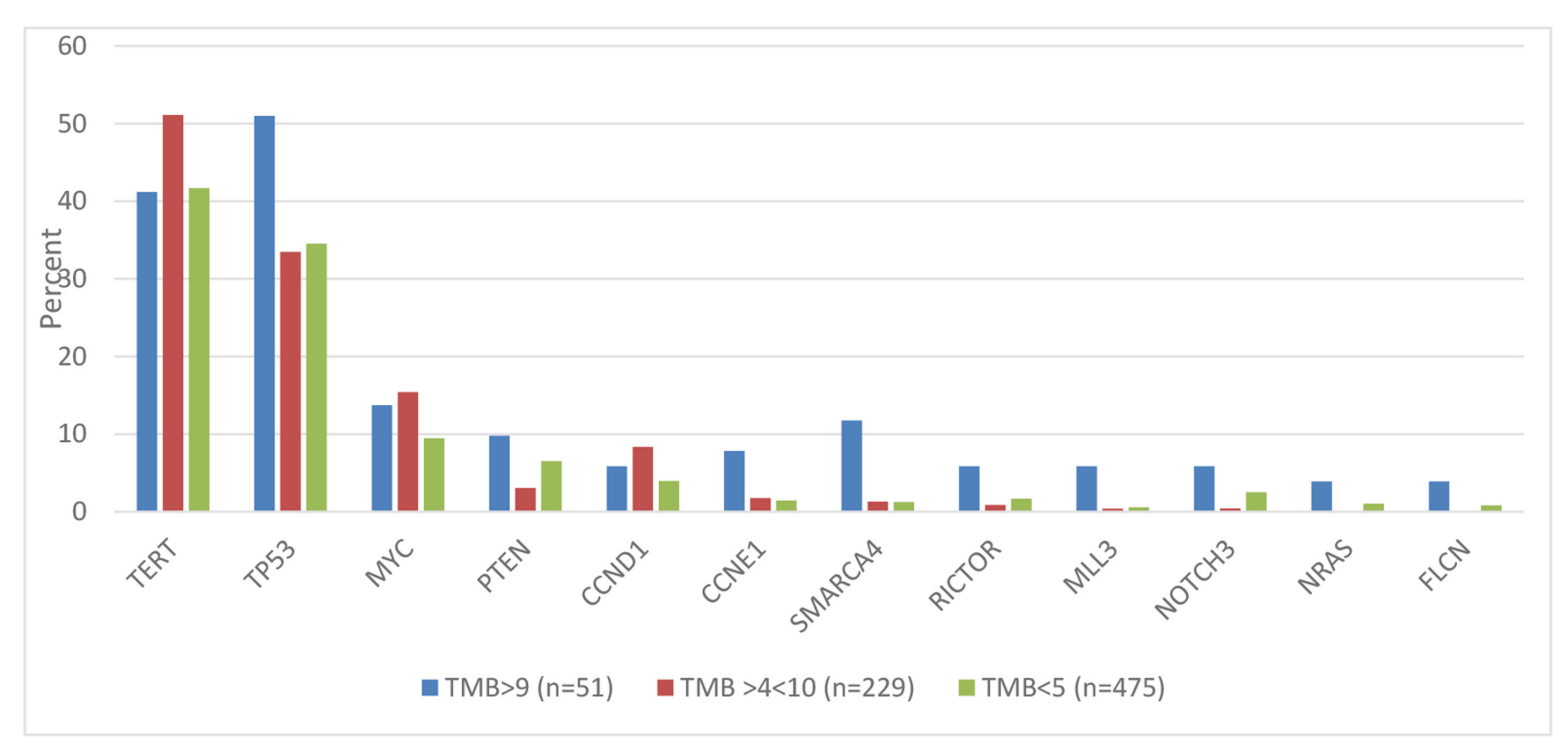
Differences in gene alteration frequencies using modified TMB cutoffs (all genes shown have statistically significant differences between 2 or 3 groups).

Since 90% of specimens had a TMB of <10 mutations/Mb, we explored potential genetic differences using modified TMB cutoffs: TMB <4 mutations/Mb (*N* = 476), 5-9 mutations/Mb (*N* = 227), > 10 mutations/Mb (*N* = 52). The pattern and frequency of alterations associated with the new subgroups generally mirrored those observed with the original subgroups. Gene alterations that significantly differed between at least 2 of the new TMB subgroups are shown (Figure 3).

### POLE and D mutations

Alterations in the proofreading domains of *POLE* and *POLD* were detected in 27 of 755 specimens (4%): 18 male and 9 female patients. A Caucasian male, 64 years’ old with cirrhosis, TMB 4 mutations/Mb and MSI-low had a pathogenic *POLE R762W* mutation. The other 26 patients had *POLE/D* VUS. Median TMB of all patients with *POLE/D* variants was 5 mutations/Mb with no statistically significant difference in *POLE/D* mutation frequency by etiology: 1/7 (14%) in patients with NASH, 2/96(2%) in patients with HBV including 4 coinfected with HCV and 6/107 (6%) in patients with HCV only.

### DNA damage response (DDR) genes

Genomic analysis included 31 genes involved in various DNA repair pathways such as *POLE/D*, *BRCA 1/2*, and *ATM* (Supplementary Table 1). Forty percent (*N* = 306) of 755 specimens contained DDR gene alterations including both pathogenic variants and VUS: 240 had one alteration, 47 had 2 alterations, 17 had 3 alterations, and 1 patient each had 4 and 5 alterations. Non DDR-altered HCCs had a significantly higher frequency of mutations in the *MYC* oncogene (14% vs. 9%, *P* = 0.05) and *KEAP1,* a regulator of oxidative stress and inflammation (4% vs. 1%, *P* = 0.02) [6]. DDR-altered tumors had non-significantly higher frequencies of mutations in *SPTA1* which encodes a molecular scaffold protein, cell cycle and intracellular signaling regulators *CDKN1B* and *IRS2* and the transcriptional regulator gene *NCOR1* (all 3% vs 1% in non DDR-altered tumors). TMB was not significantly different between DDR-altered and unaltered tumors. Furthermore, there were no significant differences in mutation profile or TMB levels between homologous recombination repair and DNA MMR pathways. Loss of heterozygosity (LOH) was evaluated as a possible mitigating factor, but fewer than 0.5% of patients exhibited LOH and no pattern with respect to any of the other parameters was identified (data not shown).

Gene alteration frequencies in tumors with *POLE/D* and DDR alterations are shown in Figure 1.

### Case series

Clinical data were available on 17 patients with advanced HCC who received a PD-1 inhibitor (Supplementary Table 2). Twelve patients were male and 10 were >65 years’ old. Four patients were African-American, 6 were Asian, 3 were Caucasian and 4 were Hispanic. Seven patients had an objective response including one complete response, 6 had disease progression, 3 patients had stable disease and one patient discontinued treatment due to toxicity before response could be assessed. Eight patients were TMB-intermediate (6-15 mutations/Mb) and 9 were TMB-low (1-5 mutations/Mb). Sixteen cases were evaluable for microsatellite status; all were MSI-low.

The patient who had a durable (>2 year) complete response had a history of HCV and the highest TMB of all patients in the series (15 mutations/Mb). Overall, TMB levels did not segregate by response; 3 TMB-low patients responded to nivolumab, and 2 with TMB-high tumors had primary progression. One patient (#6) with TMB-intermediate disease received one dose of nivolumab and subsequently developed sepsis secondary to a hepatic abscess. Nivolumab was discontinued and the patient enrolled in hospice but was discharged 4 months later due to a sustained improvement in his clinical condition. Repeat imaging indicated that he had, in fact, responded to nivolumab, and that the abscess might have developed in the context of tumor necrosis. Nivolumab was resumed for one dose but soon after he developed rapid disease progression and died one month later. Nine patients had DDR alterations: 6 patients who had a partial response or stable disease each had 1-2 alterations, 2 patients each with 2 variants had disease progression, and one patient with 4 variants received a single dose of nivolumab but was hospitalized for hepatic decompensation and discontinued treatment before response assessment. No patients had *POLE/D* alterations.

## DISCUSSION

Immune checkpoint inhibitors have revolutionized oncology treatment paradigms, but the high cost and potential for serious, albeit rare, adverse events with these agents demand judicious patient selection. Immunotherapy biomarker development has therefore become a research priority. Although PD-L1 expression is an FDA approved biomarker, its shortcomings including assay heterogeneity, variations in interpretation, and limited sensitivity for detecting responders [7, 8] has shifted attention to the concept of genomic instability as a predictor of susceptibility to PD-1 inhibitors.

The study population characteristics were reflective of the typical HCC patient profile with respect to gender ratio, age and sites of metastases. As clinical information was not available for most patients, risk factors were ascribed in only ~25% of cases, most of whom had viral hepatitis. The frequency and spectrum of genetic alterations in this cohort were consistent with prior series and expand on the genes interrogated more comprehensively [9–11].

Markers of genomic instability were generally infrequent in this cohort. Only one out of 542 evaluable specimens was MSI-high. In a recent series of 122 HCCs associated with a variety of etiologies and assessed using the gold standard pentaplex PCR technique, no tumors were MSI-high, but 26% exhibited lower levels of MSI [12]. Low level MSI tended to be more frequent in patients with cirrhosis, possibly reflecting genomic instability induced by chronic inflammation. In this series, 4 patients (0.7%) were microsatellite ambiguous (neither MSI-low nor MSI-high).

High nonsynonymous TMB is hypothesized to generate increased neoantigen expression by cancer cells [13], marking them as targets for an immune system activated by immune checkpoint inhibition. While increasing TMB correlates with higher response rates to PD-1 inhibitors [14–17], only 55% of the differences observed across tumor types are attributable to TMB [15], indicating that other factors may influence response to these agents [8]. Studies in metastatic melanoma and non-small cell lung cancer have also shown that TMB values in responders and nonresponders frequently overlap [14, 16]. Indeed, within our case series, TMB levels ranged from 3–15 mutations/Mb in patients who responded or had stable disease, and from 4–9 mutations/Mb among those who progressed.

Although the availability of targeted next generation sequencing cancer gene panels has made TMB assessment more accessible and demonstrates comparable accuracy to whole exome sequencing [16, 18, 19], the application of TMB to clinical practice remains challenging. Currently no standardized gene platform exists to calculate TMB, definitions vary across studies, and tumor heterogeneity may result in TMB estimates that do not reflect the global TMB landscape within a tumor or across the total disease burden of a patient [20]. Furthermore, TMB ranges vary across tumor types [18], suggesting that disease specific TMB definitions should be explored. One might also speculate as to whether HCC TMB differs by type and number of risk factors (ie. HCV infection only vs HCV and alcohol). In this series, no discernable difference in TMB between HCV and HBV infected patients appeared.

The genetic profile of a tumor may also help to distinguish responders from nonresponders. In a study of pembrolizumab in non-small lung cancer, a “smoking signature” characteristic of tobacco-induced mutagenesis correlated with efficacy [21]. In this case series, there were no emergent genetic signals distinguishing responders from nonresponders due to the small number of patients.

*POLE*/*D* mutations appear to define a subset of cancers with an ultra-mutated phenotype that are often MSI-low and have been linked with durable responses to PD-1 inhibition in multiple tumor types [22, 23]. Within our study cohort, only one patient had a pathogenic *POLE* R762W mutation previously identified in colorectal cancer affecting the catalytic domain of the enzyme [24]. Notably, this patient had a TMB of only 4 mutations/Mb. The remaining patients evaluable for *POLE/D* alterations had VUS which, based on the low median TMB for the entire group, suggests that these variants were not associated with clinically meaningful changes in enzyme function.

Recently, DDR alterations have also been associated with an increased TMB, tumor lymphocytic infiltration and response to PD-1 inhibition [25]. In a retrospective series of metastatic urothelial cancers, the presence of DDR alterations, especially known pathogenic alterations, was independently associated with improved clinical outcomes on PD-1/PD-L1 inhibitors [25]. DDR alterations were prevalent in this cohort of HCCs and in our case series, both responders and nonresponders had >1 DDR alteration.

The process of biomarker discovery for immunotherapeutic agents is rapidly evolving. It is increasingly recognized that complex interactions between genetic, microenvironmental and systemic factors determine response or lack of response to these agents. Existing biomarkers appear to identify small, partially overlapping and/or complementary patient subgroups that, collectively, may cast a more inclusive net to identify treatment candidates. It is plausible to conceive of an immunotherapy response prediction matrix consisting of TMB, genomic profile, cell surface protein expression and other emerging blood, stromal and even stool-based markers [26, 27] to facilitate patient selection in the future.

The main weakness of this study is the lack of clinical outcomes information for most patients precluding a more in-depth analysis of the relationships between specific biomarkers and response to PD-1 inhibition. Tumor specimens were heterogeneous with respect to site of origin and timing of procurement. The extent to which tumor genomic profiles were modified by prior treatments cannot be accounted for given the lack of clinical data. However, the consistency of our genomic profiling data with other studies [9–11] is reassuring.

In conclusion, this is the first study, to our knowledge, to evaluate the pattern and frequency of current and emerging biomarkers of response to PD-1 inhibitors in HCC. Gene alterations linked with hypermutability are infrequent in advanced HCC, and the apparent absence of an association between TMB and response to PD-1 inhibition suggests that future biomarker development in this context may need to look beyond markers of genomic instability. A follow-up study with clinical outcomes data is warranted to further elucidate determinants of response to PD-1 inhibitors in this disease.

## MATERIALS AND METHODS

Comprehensive genomic profiling (CGP) of 315 cancer-related genes was performed on 755 consecutive cases of hepatocellular carcinoma (2012-17) using a validated hybrid-capture, adaptor ligation based NGS assay (FoundationOne^®^) (Foundation Medicine, Cambridge, MA, USA; CLIA certified, CAP-accredited, NY State-approved laboratory). Pathologic diagnosis of each case was confirmed on routine hematoxylin and eosin-stained slides. A 20% minimum tumor nuclei content was required for DNA extraction, and genomic alterations (GA: point mutations, indels, copy number changes and rearrangements) were recorded (median coverage depth of 743x). Sequence analysis included hepatitis B (HBV) viral sequence reference genome alignment. Pathology reports were reviewed for documentation of HBV or hepatitis C (HCV). TMB was calculated from up to 1.1 megabase (Mb) of cancer genome as the number of somatic, coding point mutations and indels per Mb (low: <6; intermediate: 6-19; high: ≥20 mutations/Mb). Microsatellite status was determined by a novel algorithm analyzing length variability of 114 specific homopolymer repeat loci and was evaluable in 542 of the 755 patients’ tissues. PD-L1 immunostaining of 5 micron thick FFPE tissue sections was performed on the Dako Autostainer Link 48 platform and an automated staining protocol validated for the monoclonal mouse anti-human PD-L1 antibody, clone 22C3 pharmDx assay. Positive staining was defined as complete circumferential or partial cell membrane staining of viable tumor cells. Scoring was recorded as percentage of PD-L1-positive tumor cells of the total tumor cells evaluated (TPS). The specimen was considered to have PD-L1 expression if TPS ≥1% and high PD-L1 expression if TPS ≥50% [28]. Only 65 of 755 patients had PD-L1 testing performed or documented. Approval for this study, including a waiver of informed consent and HIPAA waiver of authorization, was obtained from the Western Institutional Review Board (Protocol 20152817).

## SUPPLEMENTARY MATERIALS




