# Supplementary material for: Prevalence of established and emerging biomarkers of immune checkpoint inhibitor response in advanced hepatocellular carcinoma
Source: Oncotarget. 2019 Jun 18;10(40):4018–25. doi: 10.18632/oncotarget.26998 (PMC6592287; doi:10.18632/oncotarget.26998)
Supplement: Supplementary file 1 [file oncotarget-10-4018-s001.pdf]

# Prevalence of established and emerging biomarkers of immune checkpoint inhibitor response in advanced hepatocellular carcinoma

## SUPPLEMENTARY MATERIALS

**Supplementary Table 1: DNA damage response genes tested**

| | | | | | |
|---|---|---|---|---|---|
| *APC* | *BARD1* | *CHEK2* | *MLH1* | *PARP1* | *RB1* |
| *ARID1A* | *BLM* | *FANCA* | *MRE11A* | *PMS2* | |
| *ARID2* | *BRCA1* | *FANCC* | *MSH2* | *POLD1* | |
| *ATM* | *BRCA2* | *FANCD2* | *MSH6* | *POLE* | |
| *ATR* | *BRIP1* | *FANCE* | *MUTYH* | *RAD50* | |
| *BAP1* | *CHEK1* | *KDM5A* | *PALB2* | *RAD51C* | |

**Supplementary Table 2: Case Series.** See Supplementary Table_2
