# Supplementary material for: Prevalence of established and emerging biomarkers of immune checkpoint inhibitor response in advanced hepatocellular carcinoma
Source: Oncotarget. 2019 Jun 18;10(40):4018–25. doi: 10.18632/oncotarget.26998 (PMC6592287; doi:10.18632/oncotarget.26998)
Supplement: Supplementary file 2 [file oncotarget-10-4018-s002.doc]

| **Supplementary Table 2: Case Series** | | | | | | | | | | | | | | | | | | | | | | | | | | | | | | | | | | | | | | | | | | | | | | | | | | | | | | |
| --- | --- | --- | --- | --- | --- | --- | --- | --- | --- | --- | --- | --- | --- | --- | --- | --- | --- | --- | --- | --- | --- | --- | --- | --- | --- | --- | --- | --- | --- | --- | --- | --- | --- | --- | --- | --- | --- | --- | --- | --- | --- | --- | --- | --- | --- | --- | --- | --- | --- | --- | --- | --- | --- | --- |
| **Pt** | **Risk factor** | **Age/**  **Sex** | **Ethnicity/**  **Specimen Site** | **TMB; MS status** | **Prior therapies** | **Biopsy to IO (mos)** | **Agent** | **Best response** | **Current status** | **TTF (mos)** | **Genes Altered, in order of most to least patients with alteration** | | | | | | | | | | | | | | | | | | | | | | | | | | | | | | | | | | | | | | | | **Genes altered in only 1 patient; listed by patient** | | | |
|  |  |  |  |  |  |  |  |  |  |  | TERT | CREBBP | CTNNB1 | | PREX2 | LRP1B | AXIN1 | FAT1 | TP53 | ARID1B | KEAP1 | DNMT3A | MLL | PRKDC | RUNX1T1 | LYN | MYC | SDHB | SPTA1 | AKT2 | AR | ARID1A | AXL | BRCA2 | CCND3 | EP300 | EPHB4 | FAT3 | HOXB13 | IL7R | MET | MLL2 | MLL3 | NBN | NFE2L2 | PIK3C2B | PLCG2 | POLD1 | TSC2 | VEGFA |  |  |  |  |
| 11 |  | 80 | Liver | 15;  MSi-L | sorafenib, TACE, ARQ197 | **8** | Nivo | CR | Ongoing CR, now 26 months | NA |  |  |  | |  |  |  |  |  |  |  |  |  |  |  |  |  |  |  |  |  |  |  |  |  |  |  |  |  |  |  |  |  |  |  |  |  |  |  |  | BRIP1, CD79A, CEBPA, CIC, GRM3, MAP3K1, NF1, PIK3CG | | | |
| 6 | EtOH | 68 | Liver | 12;  MSi-L | sorafenib | 4 | Nivo | PR | Deceased | 5 |  |  |  | |  |  |  |  |  |  |  |  |  |  |  |  |  |  |  |  |  |  |  |  |  |  |  |  |  |  |  |  |  |  |  |  |  |  |  |  | NTRK1, SF3B1, TSC2, VEGFA | | | |
| 2 | EtOH | 69 | Liver | 12;  MSi-L | Resection, TACE | 7 | Nivo | PR | On therapy, responding | NA |  |  |  | |  |  |  |  |  |  |  |  |  |  |  |  |  |  |  |  |  |  |  |  |  |  |  |  |  |  |  |  |  |  |  |  |  |  |  |  | BCL6, BMPR1A, CCND1, FGFR1, FRS2, KDR, LRP6, MDM2, NFKB1A, RBM10 | | | |
| 12 |  | 70 | Liver | 11;  MSi-L | Resection, TACE, ablation | 128 | Nivo | PR | On therapy, responding | NA |  |  |  | |  |  |  |  |  |  |  |  |  |  |  |  |  |  |  |  |  |  |  |  |  |  |  |  |  |  |  |  |  |  |  |  |  |  |  |  | ATM, CHD2, CYLD, DICER1, EPHA7, EZH2, FGFR2, IGF1R, IPHAS, NUP93, ZNF703 | | | |
| 3 |  | 86 | Liver | 5; MSi-L | Resection, sorafenib | 9 | Nivo | PR | On therapy, responding | NA |  |  |  | |  |  |  |  |  |  |  |  |  |  |  |  |  |  |  |  |  |  |  |  |  |  |  |  |  |  |  |  |  |  |  |  |  |  |  |  | PIK3C3, PIK3CB | | | |
| 1 |  | 68 | Liver | 5; MSi-L | Resection | 5 | Nivo | PR | Developed focal progression Tx with RT, continues nivolumab | 19 |  |  |  | |  |  |  |  |  |  |  |  |  |  |  |  |  |  |  |  |  |  |  |  |  |  |  |  |  |  |  |  |  |  |  |  |  |  |  |  | ALOX12B, CBL, FGFR3, FOX_1, GNA13, JAK1, LZTR1, PARP4, PRSS1, RANBP2 | | | |
| 13 | EtOH HIV+ | 57 | Adrenal | 3; MSi-L | none | 2 | Nivo | PR | Stopped Tx after 3 months. Alive. | NA |  |  |  | |  |  |  |  |  |  |  |  |  |  |  |  |  |  |  |  |  |  |  |  |  |  |  |  |  |  |  |  |  |  |  |  |  |  |  |  | GRM3, MCL1, NOTCH1, NOTCH3, PAX5, RUNX1T1, SPEN | | | |
| 5 |  | 60 | Liver | 8; MSi-L | TACE, Y90 | 1 | Nivo | NE | Stopped Tx for toxicity before response evaluation. Deceased. | NA |  |  |  | |  |  |  |  |  |  |  |  |  |  |  |  |  |  |  |  |  |  |  |  |  |  |  |  |  |  |  |  |  |  |  |  |  |  |  |  | ARID2, FANCE, GLI1, GNA11, IRF4, MED12, MSH6, PDGFRA, VEGFA | | | |
| 8 |  | 60 | Liver | 6; n.e. | sorafenib, experimental agent | 12 | Pembro | SD | Deceased | 7 |  |  |  | |  |  |  |  |  |  |  |  |  |  |  |  |  |  |  |  |  |  |  |  |  |  |  |  |  |  |  |  |  |  |  |  |  |  |  |  | ATR, CHEK2, CUL3, GNAS, IKZF1, IRF2, PAK3 | | | |
| 14 |  | 66 | Liver | 1; MSi-L | sorafenib | 3 | Nivo | SD | SD on nivo + regorafenib | 26* |  |  |  | |  |  |  |  |  |  |  |  |  |  |  |  |  |  |  |  |  |  |  |  |  |  |  |  |  |  |  |  |  |  |  |  |  |  |  |  |  | | | |
| 16 |  | 69 | Liver | 5; MSi-L | None, refused TKI | 11 | Pembro | SD | Deceased | 7 |  |  |  | |  |  |  |  |  |  |  |  |  |  |  |  |  |  |  |  |  |  |  |  |  |  |  |  |  |  |  |  |  |  |  |  |  |  |  |  |  | | | |
| 7 |  | 50 | Liver | 9; MSi-L | Resection, sorafenib | 1 mo post IO | Nivo | PD | Lost to follow up | 2 |  |  |  | |  |  |  |  |  |  |  |  |  |  |  |  |  |  |  |  |  |  |  |  |  |  |  |  |  |  |  |  |  |  |  |  |  |  |  |  | FANCA, INPP4B, MLH1, MPL, NUP93, PLCG2, PRKAR1A | | | |
| 9 | EtOH | 60 | Liver | 9; MSi-L | Experimental agent | 14 | Nivo | PD | Deceased | 1 |  |  |  | |  |  |  |  |  |  |  |  |  |  |  |  |  |  |  |  |  |  |  |  |  |  |  |  |  |  |  |  |  |  |  |  |  |  |  |  | CASP8, CDH5, KDM5, PLCG2, RB1, ROS1, TBX3 | | | |
| 4 | EtOH | 60 | Spine | 5; MSi-L | Resection, sorafenib + OMP54F28 | 9 | Nivo | PD | Received regorafenib, lenvatinib, cabozantinib. Now off therapy | 5 |  |  |  | |  |  |  |  |  |  |  |  |  |  |  |  |  |  |  |  |  |  |  |  |  |  |  |  |  |  |  |  |  |  |  |  |  |  |  |  | EPHB1, FLT3, STK11, TSHR | | | |
| 10 | EtOH | 63 | Liver | 5; MSi-L | sorafenib | 4 | Nivo | PD | Deceased | 2 |  |  |  | |  |  |  |  |  |  |  |  |  |  |  |  |  |  |  |  |  |  |  |  |  |  |  |  |  |  |  |  |  |  |  |  |  |  |  |  | CDKN2A, CDKN2B, FBX27, INS4, PPP2R1A | | | |
| 17 |  | 76 | Liver | 5; MSi-L | sorafenib | 15 | Nivo | PD | Deceased | 2 |  |  |  | |  |  |  |  |  |  |  |  |  |  |  |  |  |  |  |  |  |  |  |  |  |  |  |  |  |  |  |  |  |  |  |  |  |  |  |  |  | | | |
| 15 |  | 66 | Liver | 4; MSi-L | TACE, sorafenib | 5 | Nivo | PD | Deceased | 10 |  |  |  | |  |  |  |  |  |  |  |  |  |  |  |  |  |  |  |  |  |  |  |  |  |  |  |  |  |  |  |  |  |  |  |  |  |  |  |  |  | | | |
|  |  |  |  |  |  |  |  |  | *12 months on nivo only; 14 months on nivo + regorafenib | |  |  |  | |  |  |  |  |  |  |  |  |  |  |  |  |  |  |  |  |  |  |  |  |  |  |  |  |  |  |  |  |  |  |  |  |  |  |  |  |  |  |  |  |
|  |  |  |  |  |  |  |  |  |  |  |  |  |  | |  |  |  |  |  |  |  |  |  |  |  |  |  |  |  |  |  |  |  |  |  |  |  |  |  |  |  |  |  |  |  |  |  |  |  |  |  |  |  |  |
|  |  |  |  |  |  |  |  |  |  |  |  |  |  | |  |  |  |  |  |  |  |  |  |  |  |  |  |  |  |  |  |  |  |  |  |  |  |  |  |  |  |  |  |  |  |  |  |  |  |  |  |  |  |  |
|  | HCV | Female | Caucasian | TMB>6<20 |  |  |  |  |  |  | promoter -124c>t | | | | |  |  |  |  |  |  |  |  |  |  |  |  |  |  |  |  |  |  |  |  |  |  |  |  |  |  |  |  |  |  |  |  |  |  |  |  |  |  |  |
|  |  |  |  |  |  |  |  |  |  |  |  |  | |  |  |  |  |  |  |  |  |  |  |  |  |  |  |  |  |  |  |  |  |  |  |  |  |  |  |  |  |  |  |  |  |  |  |  |  |  |  |  |  |  |
|  | HBV | Male | Afr Americ | TMB<6 |  |  |  |  |  |  |  |  | |  |  |  |  |  |  |  |  |  |  |  |  |  |  |  |  |  |  |  |  |  |  |  |  |  |  |  |  |  |  |  |  |  |  |  |  |  |  |  |  |  |
|  |  |  |  |  |  |  |  |  |  |  |  |  | |  |  |  |  |  |  |  |  |  |  |  |  |  |  |  |  |  |  |  |  |  |  |  |  |  |  |  |  |  |  |  |  |  |  |  |  |  |  |  |  |  |
|  | NASH |  | Hispanic |  |  |  |  |  |  |  |  |  | |  |  |  |  |  |  |  |  |  |  |  |  |  |  |  |  |  |  |  |  |  |  |  |  |  |  |  |  |  |  |  |  |  |  |  |  |  |  |  |  |  |
|  |  |  |  |  |  |  |  |  |  |  |  |  | |  |  |  |  |  |  |  |  |  |  |  |  |  |  |  |  |  |  |  |  |  |  |  |  |  |  |  |  |  |  |  |  |  |  |  |  |  |  |  |  |  |
|  |  |  | Asian |  |  |  |  |  |  |  |  |  | |  |  |  |  |  |  |  |  |  |  |  |  |  |  |  |  |  |  |  |  |  |  |  |  |  |  |  |  |  |  |  |  |  |  |  |  |  |  |  |  |  |
